# The Association between Antihypertensive Medication Use and Blood Pressure Is Influenced by Obesity

**DOI:** 10.1155/2018/4573258

**Published:** 2018-10-01

**Authors:** Jash S. Parikh, Arshdeep K. Randhawa, Sean Wharton, Heather Edgell, Jennifer L. Kuk

**Affiliations:** ^1^York University, School of Kinesiology and Health Science, Toronto, ON, Canada M3J 1P3; ^2^The Wharton Medical Clinic, Toronto, ON, Canada M4J 5B9

## Abstract

**Introduction:**

One in three US adults is living with obesity or hypertension, and more than 75% of hypertensive individuals are using antihypertensive medications. Therefore, it is important to examine blood pressure (BP) differences in populations that are using these medications with differing obesity status.

**Aim:**

We examined whether BP attained when using various antihypertensive medications varies amongst different body mass index (BMI) categories and whether antihypertensive medication use is associated with differences in other metabolic risk factors, independent of BMI.

**Methods:**

Adults with hypertension from the National Health and Nutrition Examination Survey (NHANES) from 1999 to 2014 were used (*n*=15,285). Linear regression analyses were used to examine the main effects and interaction between antihypertensive use and BMI.

**Results:**

In general, users of antihypertensive medications had lower BP than those not taking BP medications (NoBPMed) (*P* < 0.05), whereby in women, the differences in systolic BP between angiotensin-converting-enzyme (ACE) inhibitor or angiotensin receptor blocker (ARB) users and NoBPMed were greater in those with obesity (ACE inhibitors: −14 ± 1 mmHg; ARB: −16 ± 1 mmHg) compared to normal weight individuals (ACE inhibitors: −9 ± 1 mmHg; ARB: −11 ± 1 mmHg) (*P* < 0.05). Diastolic BP differences between women ARB users and NoBPMed were also greatest in obesity (−5 ± 1 mmHg) (*P* < 0.05) whilst there were no differences in normal weight individuals (−1 ± 1 mmHg) (*P*>0.05). Furthermore, glucose levels and waist circumference in women were higher in those using ACE inhibitors compared to diuretics (*P* < 0.05).

**Conclusion:**

ACE inhibitors and ARBs may be associated with more beneficial BP profiles in women with obesity, with no obesity-related BP differences for antihypertensive medication in men. However, there could be potential cardiometabolic effects for some antihypertensive medications that should be explored further.

## 1. Introduction

Cardiovascular disease (CVD) is now recognized as the leading cause of death worldwide [1]. In 2015, more than 17 million deaths were reported due to CVD and is expected to grow to more than 23 million by 2030 [2]. Hypertension increases the risk of stroke by almost three-fold and is amongst the top 15 leading causes of death in the United States [3]. Obesity is a risk factor for hypertension [4, 5], and over the past few decades, the rates of obesity have risen dramatically [6]. Considering the independent and combined CVD risks of obesity and hypertension, these chronic conditions are major public health issues.

The prevalence of antihypertensive medication use in US adults with hypertension has risen from 64% to 77% in 2001 to 2010 [7]. Overall, the use of first-line antihypertensive medications including thiazide diuretics, *β*-blockers, angiotensin-converting-enzyme (ACE) inhibitors, or angiotensin receptor blockers (ARBs) has increased by 23%, 57%, 31%, and 100%, over the same time frame [7]. However, there are many differences in the physiology of obesity-related hypertension that may impact the effectiveness of antihypertensive medications in populations with varying obesity. Moreover, differences in body composition have been reported to influence the pharmacokinetics of various medications [8, 9]. Further, individuals with obesity are reported to be less adherent to taking medications than lean individuals [10]. It is also well known that medication adherence in clinical trials are far superior to real-world settings [11]. Thus, with the substantial rise in antihypertensive medication use combined with the high obesity prevalence, it is important to examine whether medication-related blood pressure differences are influenced by obesity.

There are few studies that have directly compared blood pressure differences in antihypertensive medication users across different body mass index (BMI) categories (normal weight, overweight, and obesity) [12–14]. The INVEST (International Verapamil SR-Trandolapril) trial and a post hoc analysis of the ACCOMPLISH (Avoiding Cardiovascular Events through Combination Therapy in Patients Living with Systolic Hypertension) trial in particular found that individuals with obesity on calcium channel blockers (CCBs) and diuretics in combination with ACE inhibitors were at lower risk for experiencing a cardiovascular event than their lean counterparts [12, 13]. To date, no studies have compared blood pressure differences between different BMIs for all of the common first-line antihypertensive classes [11–14]. Furthermore, past literature has only examined the individual blood pressure effects of the antihypertensive medication types in combination with other classes [13–16], and thus the individual medication effects becomes difficult to assess. In addition, none of these studies observed the association between antihypertensive medication use and metabolic risk factors. Therefore, the objective of this study was to determine whether blood pressure attained using various antihypertensive medications is different amongst individuals with various BMI. This study also determined whether the use of antihypertensive medications is associated with differences in glucose and lipid levels and waist circumference independent of BMI.

## 2. Methods

### 2.1. Participants

The National Health and Nutrition Examination Survey (NHANES) is a series of health surveys conducted by the Centers for Disease Control and Prevention (CDC) and the National Center for Health Statistics (NCHS). NHANES utilizes a cross-sectional, multistage, probability study design to select participants that are nationally representative of the civilian, noninstitutionalized US population by oversampling African Americans, Mexican Americans, and individuals aged 60 years or older. The survey consists of an in-home interview, followed by a standardized health examination and laboratory tests in mobile examination centers (MECs). All participants provided their informed written consent for the in-home interview and physical examination, and ethics were approved by the NCHS Research Ethics Review Board. Further information on the study protocol and design is reported by CDC [17].

NHANES continuous data (1999–2014) were used (*n*=82,091). Participants were excluded from analysis if they were under the age of 18 years old (*n*=34,735), if they had a BMI < 18.5 kg/m^2^ (*n*=653), and if they answered “Yes” to “Are you now taking prescribed medicine for high blood pressure?” but had no blood pressure medication data (*n*=588). Additionally, participants were excluded if they had missing data for BMI (*n*=3,246), health insurance status (*n*=347), smoking status (*n*=3,067), physical activity (*n*=5), education (*n*=104), systolic blood pressure (SBP; *n*=4,380), and diastolic blood pressure (DBP; *n*=4,611). The final sample size was 37,763 of which 15,285 were hypertensive (defined as self-reported doctor-diagnosed hypertension or SBP ≥ 140 mmHg or DBP ≥ 90 mmHg or self-reported antihypertensive medication use) and used for analysis.

Any additional missing data for analysis of fasting plasma glucose (FPG), waist circumference (WC), triglyceride (TG), and high-density lipoprotein (HDL) were excluded (FPG: *n*=20,177; WC: *n*=1,061; TG: *n*=20,325; HDL: *n*=1,969). The final sample size for analysis was 7,146 for FPG, 14,691 for WC, and 6,731 for HDL and TGs.

### 2.2. Survey Methods

#### 2.2.1. Household Questionnaires

Age, gender (male/female), ethnicity (white/others), health insurance status (insured/uninsured), education status (<high school/≥ high school), physical activity (active/inactive), and smoking status (smoker/nonsmoker) were assessed through household questionnaires. Participants were considered insured if they answered “Yes” to “Are you covered by health insurance or some kind of health care plan? (including employment, private, and government health care plans).” Participants who answered “Yes” to performing moderate or vigorous physical activity leading to small or large increases in breathing and heart rate for at least 10 minutes continuously in the past 30 days were considered active. Participants that were current smokers were coded as smokers.

#### 2.2.2. Examination Measures

All body measures were obtained by trained health technicians at the mobile examination center (MEC). Standing height was measured to the nearest tenth of a centimeter (0.1 cm) using a stadiometer with a fixed vertical backboard and an adjustable head piece. Weight was measured in kilograms using a digital weight scale. BMI was then calculated using weight in kilograms divided by height in meters squared (kg/m^2^). Participants were categorized as normal weight (18.5 kg/m^2^ ≤ BMI < 25 kg/m^2^), overweight (25 kg/m^2^ ≤ BMI < 30 kg/m^2^), or obesity (BMI ≥ 30 kg/m^2^). WC was measured by extending the measuring tape around the waist at the participant's uppermost lateral border of the right ilium. WC was measured to the nearest tenth of a centimeter (0.1 cm). FPG and TG levels were assessed on participants who were examined in the morning session after a minimum eight hours fast (NHANES 1999–2004) or nine hours fast (NHANES 2005–2014).

Blood pressure measurements were obtained using a manual mercury sphygmomanometer. Prior to blood pressure measurements, upper arm circumference was measured to guide selection of cuff sizes. Further details on the protocol for obtaining upper arm circumference are described in the *Physician Examination Procedures Manual* [18]. The blood pressure cuff was placed on the right arm unless a specific condition was reported prohibiting use of the right arm. Participants were seated for 5 minutes and were told to rest quietly before beginning blood pressure measurements. At minimum, three measurements were recorded with 30 seconds rest in between each measurement. If necessary, a fourth measurement was recorded. Blood pressure measurements were averaged and used for analysis. All measurements were performed on the same day.

### 2.3. Prescription Medication Use

Prescription medication information was obtained through household questionnaires. Participants who had used prescription medication in the past 30 days were asked to provide the medication name and container to the examiner. NHANES uses a prescription medication database, Lexicon Plus®, that sorts medications by drug ingredients and therapeutic categories. Blood pressure medications were categorized as ACE inhibitors (*n*=4,609), *β*-blockers (*n*=4,243), diuretics (*n*=5,054), CCBs (*n*=3,387), ARBs (*n*=2,279), and others (alpha blockers, renin inhibitors, and vasodilators, *n*=974). Combination therapy was considered if participants were using ≥2 different types of antihypertensive medications. The no BP drug group (NoBPMed) included individuals with hypertension but were not taking antihypertensive medications (*n*=5,443).

### 2.4. Statistical Analysis

Participant characteristics were stratified by antihypertensive medication drug type. All analyses of antihypertensive drug types were compared to NoBPMed (hypertensive adults without reported antihypertensive medication use). Data were presented as means (SE) or prevalence, % (SE). The differences between sample characteristics were evaluated using one-way analysis of variance (ANOVA) with Tukey's post hoc tests for continuous variables and chi-square tests for categorical variables.

Linear regression analyses were used to assess the individual and joint associations of BMI category and antihypertensive medication use on blood pressure adjusted for age, gender, ethnicity, education status, health insurance status, physical activity, smoking status, and number of antihypertensive medications. Predicted least squared mean blood pressure was computed for each BMI category-antihypertensive use group. Predicted least squared means were computed for FPG, WC, TG, and HDL levels adjusted for age, gender, BMI, ethnicity, education status, health insurance status, physical activity, smoking status, and type 2 diabetes (FPG only). Differences were evaluated using Tukey's multiple comparison tests. Figures for waist and HDL were stratified by gender due to the well-known gender differences. For each analysis, gender-drug-BMI interactions were examined. When significant, the linear regression and least squared mean results were presented for men and women separately. Odds ratio for attaining blood pressure control (<140/90 mmHg) between BMI categories was assessed using logistic regression.

All analyses were performed using SAS version 9.4 (SAS Institute Inc., Cary, NC, USA) and weighted to be representative of the US population. Statistical significance was defined as *P* value <0.05.

## 3. Results

### 3.1. Participant Characteristics

Participant characteristics are shown in Table 1. In general, individuals using antihypertensive medication were older, were more likely to be women, white, have health insurance and obesity, and were less likely to smoke and be physically active than individuals not using antihypertensives (*P* < 0.05). Individuals taking antihypertensive medication had significantly lower SBP and DBP compared to NoBPMed (*P* < 0.05). With a therapeutic goal of <140/90 mmHg, the blood pressure control rate for overall antihypertensive users was 68.4%, where individuals with obesity were more likely to have controlled blood pressure, compared to normal weight users (70.4% vs. 63.3%, OR, 95% CI: 1.4, 1.2–1.6).

### 3.2. Association between BMI Category and Antihypertensive Medication Use

#### 3.2.1. Combination Therapy

**Figure 1:**
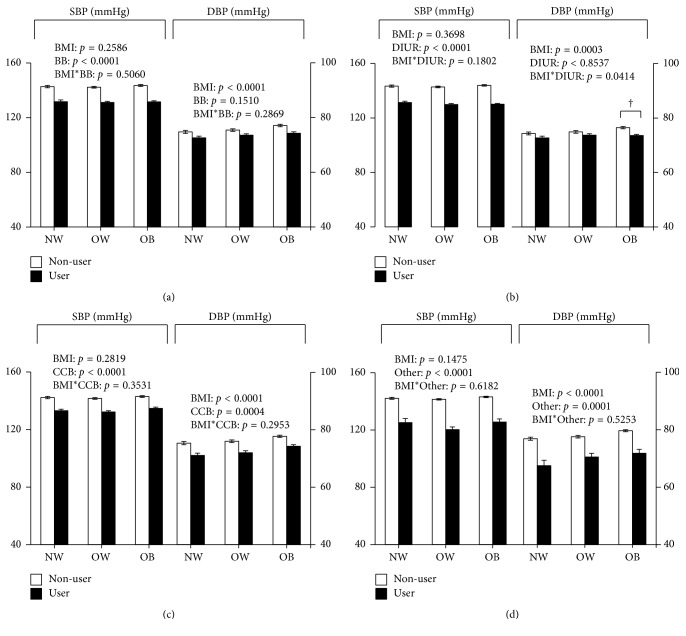
Mean systolic and diastolic blood pressures (mmHg) among hypertensive adults using (a) *β*-blockers, (b) diuretics, (c) CCBs, and (d) other antihypertensive drugs by BMI category. Sample includes individuals on combination antihypertensive therapy. Means are adjusted for age, gender, ethnicity, health insurance status, smoking status, physical activity, education, and number of antihypertensive medications taken. BMI = body mass index; BB = *β*-blocker; DIUR = diuretic; CCB = calcium channel blocker; NW = normal weight; OW = overweight; OB = obesity; SBP = systolic blood pressure; DBP = diastolic blood pressure. ^*∗*^Difference between users and nonusers significantly different from normal weight (*P* < 0.05); ^†^ difference between users and nonusers significantly different from overweight (*P* < 0.05).

There was no interaction effect observed between BMI category and *β*-blocker, CCB, or other antihypertensive use (alone or in combination) on SBP and DBP in adults with hypertension (Figure 1, *P* > 0.05). However, there was a significant interaction between antihypertensive medication use and BMI category on DBP in those using diuretics (*P*=0.0414). The difference in DBP between diuretic users and NoBPMed was slightly greater in those with obesity compared to those with overweight (−2 ±1 mmHg versus −1 ± 1 mmHg, *P*=0.0198). Diuretic users had no significant differences in SBP by BMI category (*P*>0.05). There were no additional gender interactions.

**Figure 2:**
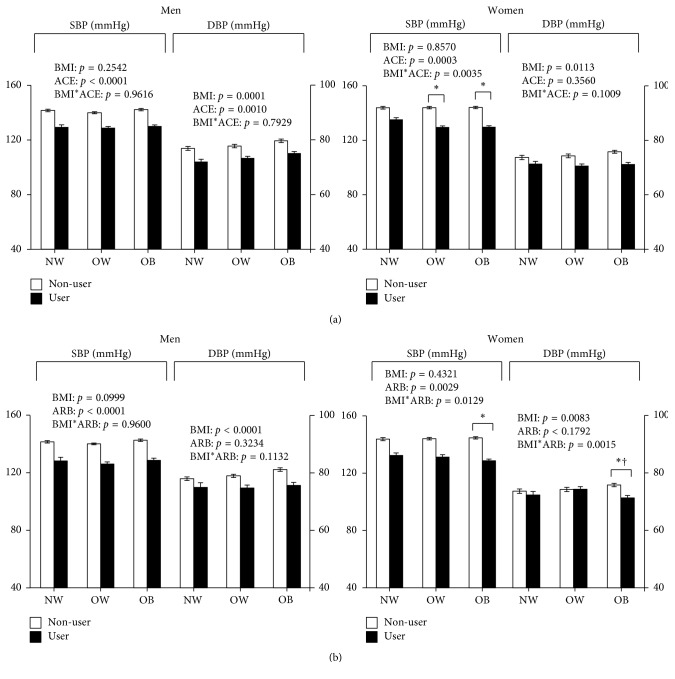
Mean blood pressures (mmHg) among hypertensive adults using (a) ACE Inhibitors and (b) ARBs by BMI category and gender. Sample includes individuals on combination antihypertensive therapy. Means are adjusted for age, ethnicity, health insurance status, smoking status, physical activity, education, and number of antihypertensive medications taken. BMI = body mass index; ACE = ACE inhibitor; ARB = angiotensin receptor blocker; NW = normal weight; OW = overweight; OB = obesity; BP = blood pressure; SBP = systolic blood pressure; DBP = diastolic blood pressure. ^*∗*^Difference between users and nonusers significantly different from normal weight (*P* < 0.05); ^†^difference between users and nonusers significantly different from overweight (*P* < 0.05).

There were significant interaction effects between antihypertensive medication use, BMI, and gender on blood pressure for ACE inhibitors and ARBs (Figure 2; ACE inhibitors SBP: *P*=0.004; ARB SBP: *P*=0.01; ARB DBP: *P*=0.002). In women, the difference in SBP between ACE inhibitor users and NoBPMed was significantly greater in those with obesity and overweight compared to normal weight (obesity versus normal weight: −14 ± 1 mmHg versus −9 ± 1 mmHg, *P*=0.0004; overweight versus normal weight: −14 ± 1 mmHg versus −9 ± 1 mmHg, *P*=0.001). The difference in SBP and DBP between ARB users and NoBPMed was significantly greater in those with obesity compared to normal weight (SBP: −16 ± 1 versus −11 ±1 mmHg, *P*=0.03; DBP: −5 ± 1 versus −1 ± 1 mmHg, *P*=0.03) and overweight (DBP: −5 ± 1 versus +1 ± 1 mmHg, *P*=0.03). Conversely, there were no effects of ACE inhibitor or ARB use by BMI on blood pressure in men (*P*>0.05).

#### 3.2.2. Monotherapy

After restricting analysis to individuals on only one type of antihypertensive medication, we observed a similar interaction effect between ACE inhibitor use and BMI on blood pressure in women (SBP: *P*=0.006; DBP: *P*=0.001) (Table 2). The difference in SBP and DBP between ACE inhibitor users and NoBPMed was significantly greater in those with obesity compared to normal weight (SBP: −15 ± 1 mmHg versus −7 ± 1 mmHg, *P*=0.002; DBP: −7 ±1 mmHg versus −2 ± 1 mmHg, *P*=0.002). However, in comparison to the earlier analysis of the antihypertensive medications types in combination which showed no interaction effect between CCB use and BMI, the restricted analysis found that the difference in SBP between CCB users and nonusers was significantly smaller in those with obesity compared to overweight (−6 ± 1 mmHg versus −11 ±1 mmHg, *P*=0.009) and normal weight individuals (−6 ±1 mmHg versus −11 ± 1 mmHg, *P*=0.03). No other differences in the restricted analysis were observed.

### 3.3. Association between Antihypertensive Medication Use and Metabolic Risk Factors

**Figure 3:**
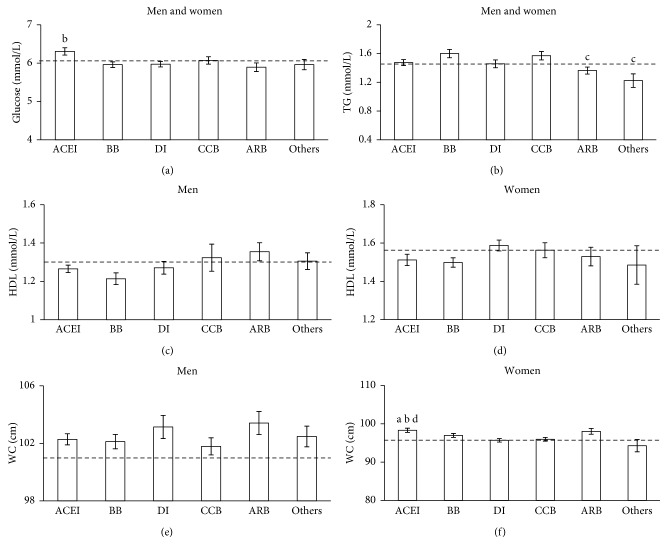
Adjusted means for cardiometabolic risk factors by antihypertensive medication type. All models adjusted for age, gender, BMI, education, health insurance, smoking, and physical activity levels. Further adjustment for type 2 diabetes performed on FPG. ACE = ACE inhibitor; BB = β-blocker; DI = diuretics; CCB = calcium channel blocker; ARB = angiotensin receptor blocker; TG = triglyceride; HDL = high-density lipoprotein; WC = waist circumference. a = significantly different from No BP Drug (*P* < 0.05); b = significantly different from diuretics (*P* < 0.05); c = significantly different from BB (*P* < 0.05); d = significantly different from CCB (*P* < 0.05). Dashed line is the no BP drug group.

Overall, there were no BMI-drug interactions (*P*>0.05) and there were few differences observed between the use of all antihypertensive medications and non-BP CVD risk factors. Thus, the differences in mean FPG, TG, HDL and WC levels by antihypertensive medication use type are shown in Figure 3. FPG levels were significantly higher in those using ACE inhibitors compared to diuretics (*P*=0.01), even when analysis was restricted to individuals with diabetes (data not shown, *P*=0.007). Triglyceride levels were lower in ARB users and the other antihypertensive medications compared to those using *β*-blockers (ARB: *P*=0.03; others: *P*=0.02). In women, WC was significantly higher with ACE inhibitor use compared to diuretics (*P*=0.004), CCBs (CCB: *P*=0.03), and NoBPMed (*P*=0.002). Neither HDL levels in men and women nor WC in men differed by antihypertensive medication type (*P*>0.10).

## 4. Discussion

Although it is clear that all antihypertension medications are associated with reductions in blood pressure, to our knowledge, this is the first study that demonstrated ACE inhibitors and ARBs were associated with bigger differences in blood pressure in women with obesity compared to lean women, while there were minimal differences in men and for other medication types. Further, we observed higher glucose and waist circumference levels in women with ACE inhibitor use compared to diuretic medication use. Thus, there may be certain antihypertensive medications that are better suited for women with obesity, which may be expected given the various mechanisms of action for antihypertensive medication and the known physiological differences by gender and obesity.

The etiology of hypertension differs widely amongst individuals with obesity as a variety of mechanisms directly involved in blood pressure regulation including the sympathetic nervous system (SNS), the renin-angiotensin aldosterone system (RAAS), and fluid balance are altered [19, 20]. Thus, there may be differences in the expected effectiveness of antihypertensive medications in those with obesity as compared to other BMI categories. We observed that women with obesity had bigger differences in blood pressure when using ACE inhibitors and ARBs than normal weight and overweight women which may be expected given the known physiology of ACE inhibitors and obesity [21–23]. Estrogen has cardioprotective effects in part through decreasing blood pressure by downregulating ACE leading to decreased angiotensin II production [24–26]. Conversely, obesity leads to an overexpression of angiotensinogen and increased blood pressure [27, 28]. Individuals with obesity may also exhibit cardioprotection due to higher circulating estrogen (a potent vasodilator) from the conversion of androgens by aromatase in adipose tissue [29]. This increase in estrogen concentration could contribute to cardiovascular protection which is known to exist in premenopausal women [30–33]. Thus, ACE inhibitors and ARBs may have the greater potential to improve hypertension for women than men, particularly in those with obesity. Although men with obesity have been shown to have elevated plasma concentrations of estrone sulfate compared to men without obesity [34], the superior blood pressure effects of ACE in obesity were only seen in women. Reasons for this are unclear, but reinforce that there may be sex differences in blood pressure regulation and pharmacological interventions that warrant more research. These results are consistent with the “obesity paradox” which suggests that obesity is associated with decreased morbidity and mortality [12, 35, 36]. Given that hypertension is one of the most important risk factors for premature CVD [37], the superior antihypertensive effect of medications in obesity may play a protective role in risk reduction. Indeed, the INVEST trial found that among patients using ACE inhibitors in combination with CCBs with a history of coronary heart disease and class I obesity had a 48% lower risk of CV-related mortality, compared to normal weight patients [12]. Therefore, ACE inhibitor use may contribute to the obesity paradox, wherein CVD morbidity and mortality is lowered in those with obesity as a result of superior blood pressure reduction.

We also observed that those taking diuretics with obesity had subtly larger differences in blood pressure profiles compared to overweight. However, there may be a physiological basis for why diuretics may be preferred in obesity [13, 38, 39]. Obesity is associated with increased sodium retention and is often the hallmark of hypertension in obesity [39]. This is due to the lower rate of natriuresis (urinary sodium excretion) because of reduced natriuretic peptides [40, 41]. Since diuretics lower blood pressure by promoting natriuresis and subsequently a reduction of plasma volume, they are a logical treatment for hypertension in those with obesity [13]. The diuretic class also includes aldosterone antagonists, and in addition to a lower rate of natriuresis, aldosterone levels are increased in obesity [42–44]. Furthermore, a subanalysis of the ACCOMPLISH trial found that patients with obesity taking a combination of ACE inhibitors and diuretics were 39%, 60%, and 40% less likely to experience primary CVD morbidity outcomes, CVD deaths, and total stroke, respectively, when compared to normal weight individuals taking the same medications. Together this may explain in part why thiazide diuretics may offer more cardiovascular protection in patients with obesity than those who are of lower BMI and that they should be preferred for first-line therapy [13]. However, obesity is known to be associated with impairments in kidney function [45] and thus may explain why we observed minimal BMI-related differences in blood pressure with diuretic use. Therefore, despite our findings, diuretics may still be a reasonable antihypertensive medication choice particularly for those with obesity.

Hypertension in individuals with obesity is commonly associated with increased sympathetic activity, often resulting in increased cardiac output (CO) [46]. CO is increased through activation of *β*-adrenergic receptors expressed in the heart causing greater heart rate and/or contractility of cardiac tissue. Thus, these effects can be antagonized by *β*-blockade. Contrary to the current findings, some studies have shown blood pressure is more sensitive to adrenergic blockade in obesity than lean individuals which may be why studies show greater blood pressure reduction with *β*-blockers in those with obesity than lean populations [47, 48]. Indeed, the concentration of *β*-adrenergic receptors have been shown to be elevated in obesity and decreased with weight loss, implying greater activity of *β*-receptors in obesity [49]. However, we and others observe that there are similar differences in BP with *β*-blocker use across BMI [8, 50]. Studies that observed an interaction between *β*-blocker effectiveness and obesity used different types of *β*-blockers (metoprolol (*β*1 selective) and atenolol (*β*1 selective) with doxazosin (α-antagonist)) compared to the studies that found no interaction (nebivolol (*β*1 blocker with nitric-oxide mediated vasodilation), propranolol (nonselective *β*-blocker), or atenolol alone). Alternatively, the lack of effect observed in our work, despite demonstrated obesity differences in the efficacy of *β*-blockers, could be attributed to differences in adherence or side effects of this drug with those with obesity. Thus, more research is needed to determine whether there are obesity-related differences in BP attained using *β*-blockers and whether there are differential effects between the specific drug types.

We observed that CCBs in combination with other medications were not associated with BP differences by obesity status. This finding is in line with the ACCOMPLISH subanalysis that suggests that cardiovascular protection, in addition to blood pressure lowering does not differ by BMI in CCB users who were concurrently using ACE inhibitors [13]. However, when we restricted the sample to those who were using CCBs alone, we observed smaller BP differences with CCB use in those with obesity as compared to lean which is in accordance with studies that have shown CCB use to be more effective in lean individuals than obesity due to the major hemodynamic action in reducing vascular resistance [47]. Physiologically, this may be logical if individuals with obesity have a greater number of blood vessels owing to their larger mass, and they may also have more numerous calcium channels requiring higher doses of CCBs in order to observe similar reductions of vascular resistance as normal weight individuals. Further, obesity is also known to be associated with smooth muscle dysfunction [51] which could be inhibiting the action of CCBs. In conjunction with the superior blood pressure lowering effects in obesity with ACE inhibitors that we observed, the discordant antihypertension medication effects in those with obesity may have been masked in the ACCOMPLISH trial. However, more work in this area is needed to clarify differences in CCB effectiveness by obesity status.

Our study reported higher glucose and waist circumference levels (in women only) with ACE inhibitor use compared to diuretics. This observation does not support studies that suggest ACE inhibitors have beneficial effects on glucose metabolism, lipid profiles, and insulin sensitivity, making them particularly attractive for first-line therapy [52, 53]. ACE inhibitors have been shown to improve glucose metabolism via translocation of the glucose transporters to the cell surface to increase glucose uptake and therefore cause a reduction of plasma glucose [54]. Thus, the differences we observed may be explained in part by the more prevalent ACE inhibitor use, but less frequent use of diuretics in individuals with diabetes. However, these observations remained similar even when the analysis was restricted to individuals with diabetes. Indeed, our results could simply be suggesting that medical professionals are prescribing ACE inhibitors more frequently to women with metabolic impairments due to the previously mentioned positive effects. Alternatively, there could be an unknown interaction between the different medications. In addition to higher glucose, we also observed higher waist circumference levels in women using ACE inhibitors compared to diuretics. Diuretics promote diuresis which can lead to weight loss/smaller waist circumference with increases in dose [55]. Furthermore, a study by Patil et al. [56] found that individuals that were given ACE inhibitors had an increased waist-hip ratio after 6 months, but did not provide a mechanism behind this observation nor did they examine metabolic differences [56]. However, the greater weight gain over time associated with ACE inhibitor use may be related to improved glucose uptake [57]. Long-term, greater insulin sensitivity could promote greater weight gain, leading to the higher plasma glucose observed. Thus, ACE inhibitor use may be associated with differential changes in metabolic risk factors with long-term use. Alternatively, there may be differences in the effect of ACE inhibitor use alone and in combination with other medications that needs further examination.

This study had strengths and limitations. The use of NHANES data allows for a large analytic sample that is nationally representative of the US population [17]. Additionally, prescription medication, examination, and blood pressure data were collected through many data collection methods including personal interviews and questionnaires which have been shown to reduce nonresponse bias when both data collection modes are used [58]. Another strength was the adjustment of many confounding variables associated with blood pressure and medication use. Although the blood pressure medications included in the sample are first-line antihypertensive medications, they are also prescribed to treat other conditions and the specific reason for prescription could not be determined nor could adherence. Furthermore, we were not able to account for dose-related effects; however, we did adjust for the number of antihypertensive medications taken. Finally, we only examined the blood pressure attained using cross-sectional survey data and did not examine changes in blood pressure; thus, if there were BMI differences in blood pressure prior to treatment, this may have confounded our results. However, as blood pressure is positively associated with BMI, we may underestimate the BMI differences in drug effectiveness by examining blood pressure attained cross sectionally. Nevertheless, as the blood pressure attained is arguably more important than the magnitude of blood pressure reduction, these results which are weighted to be nationally representative of the US population demonstrate that certain antihypertensive medications may have clinical impacts on blood pressure and other metabolic risk factors in those with and without obesity.

## 5. Conclusions

Our study suggested that ACE inhibitors and ARBs may be associated with a greater BP reduction in women with obesity compared to normal weight women, with no differences in BP between antihypertensive medication use by obesity in men. However, we did observe elevated glucose and WC levels (in women) with ACE inhibitor use in comparison to diuretics. Therefore, obesity status, in addition to potential side effects of antihypertensive medications on cardiometabolic risk, should be considered. A longitudinal examination of the long-term effectiveness of antihypertensive medications on blood pressure, cardiometabolic risk factors, and morbidity and mortality risk to determine the most appropriate choice of treatment for those with and without obesity.

## Figures and Tables

**Table 1:** Descriptive characteristics of US adults with hypertension by antihypertensive medication type (NHANES 1999–2014).

Participant characteristics	No BP drug	ACE inhibitors	*β*-blockers	Diuretics	CCB	ARB	Others
	(*n*=5443)	(*n*=4609)	(*n*=4243)	(*n*=5054)	(*n*=3387)	(*n*=2279)	(*n*=974)

Age, years	49.0 (0.3)	61.0 (0.3)^*∗*^	63.3 (0.3)^*∗*^	62.4 (0.3)^*∗*^	63.6 (0.3)^*∗*^	62.5 (0.4)^*∗*^	66.8 (0.5)^*∗*^
Gender (% female)	45.3 (0.8)	48.0 (0.9)^*∗*^	53.5 (1.0)^*∗*^	62.8 (0.8)^*∗*^	53.4 (1.1)^*∗*^	57.4 (1.3)^*∗*^	28.4 (1.9)^*∗*^
Ethnicity (% white)	69.0 (1.5)	74.6 (1.3)^*∗*^	79.3 (1.1)^*∗*^	74.9 (1.3)^*∗*^	68.9 (1.6)	74.4 (1.6)	73.5 (1.9)^*∗*^
Education (% HS or more)	79.7 (0.8)	77.3 (1.0)^*∗*^	78.0 (1.0)	76.1 (0.9)	74.8 (1.0)^*∗*^	80.7 (1.0)^*∗*^	71.6 (2.2)^*∗*^
Health insurance (% yes)	78.8 (0.8)	92.6 (0.5)^*∗*^	93.8 (0.4)^*∗*^	93.6 (0.6)^*∗*^	94.2 (0.5)^*∗*^	95.0 (0.6)^*∗*^	94.7 (0.9)^*∗*^
Smoking status (% smoker)	25.3 (0.9)	16.0 (0.7)^*∗*^	15.2 (0.8)^*∗*^	12.8 (0.7)^*∗*^	14.4 (0.9)^*∗*^	10.4 (0.9)^*∗*^	12.6 (1.5)^*∗*^
Physical activity (% active)	55.2 (1.0)	46.2 (1.1)^*∗*^	45.9 (1.1)^*∗*^	43.9 (1.3)^*∗*^	45.0 (1.0)^*∗*^	47.5 (1.7)^*∗*^	45.3 (2.2)^*∗*^

*Body mass index (%)*							
Normal weight	24.3 (0.7)	16.0 (0.6)^*∗*^	18.5 (0.7)^*∗*^	14.5 (0.6)^*∗*^	18.5 (0.8)^*∗*^	14.1 (0.9)^*∗*^	15.8 (1.4)^*∗*^
Overweight	35.4 (0.8)	33.6 (0.9)	34.9 (0.9)	30.6 (0.9)^*∗*^	32.6 (0.9)^*∗*^	31.7 (1.4)^*∗*^	33.5 (1.7)
Obesity	40.3 (0.9)	50.4 (0.9)^*∗*^	46.6 (0.9)^*∗*^	54.9 (1.0)^*∗*^	48.9 (1.0)^*∗*^	54.2 (1.4)^*∗*^	50.7 (1.8)^*∗*^

*Blood pressure (mmHg)*							
Systolic	138 (1)	130 (1)^*∗*^	132 (1)^*∗*^	131 (1)^*∗*^	135 (1)^*∗*^	133 (1)^*∗*^	133 (1)^*∗*^
Diastolic	79 (1)	70 (1)^*∗*^	70 (1)^*∗*^	70 (1)^*∗*^	69 (1)^*∗*^	70 (1)^*∗*^	68 (1)^*∗*^
% control	39.8 (1.1)	70.6 (0.9)^*∗*^	66.7 (0.9)^*∗*^	68.7 (0.8)^*∗*^	62.5 (1.1)^*∗*^	65.9 (1.2)^*∗*^	66.0 (1.8)^*∗*^
Number of BP meds	--------	2.0 (0.1)	2.3 (0.1)	2.4 (0.1)	2.4 (0.1)	2.3 (0.1)	2.8 (0.1)

^*∗*^Significantly different from the no BP drug group (*P* < 0.05). Continuous data presented as means (SE), categorical data presented as prevalence, % (SE), estimates weighted to represent the US population. Antihypertensive drug groups are not mutually exclusive. ACE = angiotensin-converting enzyme; CCB = calcium channel blocker; ARB = angiotensin receptor blocker; BP = blood pressure; HS = high school; BMI = body mass index; normal weight = 18.5 kg/m^2^ < BMI < 25 kg/m^2^; overweight = 25 kg/m^2^ ≤ BMI < 30 kg/m^2^; obesity = BMI ≥ 30 kg/m^2^; SE = standard error.

**Table 2:** Blood pressure differences between users and nonusers of individuals on antihypertensive monotherapy by BMI category.

	Normal weight		Overweight		Obesity	
	Sample size *N* (%)	BP diff (mmHg)	Sample size *N* (%)	BP diff (mmHg)	Sample size *N* (%)	BP diff (mmHg)
*SBP (mmHg)*						
ACE inhibitors	305 (17.8)	**M:** −16 (1) **W:** −7 (1)	548 (35.4)	**M:** −11 (1) **W:** −14 (1)^*∗*^	648 (46.8)	**M:** −12 (1) **W:** −15 (1)^*∗*^
Β-blockers	252 (24.1)	−16 (1)	408 (38.6)	−10 (1)	383 (37.3)	−11 (1)
Diuretics	178 (19.3)	−16 (1)	267 (31.6)	−14 (1)	408 (49.1)	−14 (1)
CCB	189 (24.1)	−11 (1)	284 (34.2)	−11 (1)	291 (41.7)	−6 (1)^*∗*^^**†**^
ARB	103 (17.9)	**M:** −15 (1) **W:** −12 (1)	179 (34.1)	**M:** −14 (1) **W:** −14 (1)	245 (48.0)	**M:** −12 (1) **W:** −15 (1)
Others	47 (27.8)	−20 (1)	73 (36.4)	−20 (1)	57 (35.8)	−17 (1)

*DBP (mmHg)*						
ACE inhibitors	305 (17.8)	**M:** −8 (1) **W:** −2 (1)	548 (35.4)	**M:** −7 (1) **W:** −4 (1)	648 (46.8)	**M:** −6 (1) **W:** −7 (1)^*∗*^^**†**^
β-blockers	252 (24.1)	−7 (1)	408 (38.6)	−4 (1)	383 (37.3)	−5 (1)
Diuretics	178 (19.3)	−4 (1)	267 (31.6)	−5 (1)	408 (49.1)	−6 (1)
CCB	189 (24.1)	−7 (1)	284 (34.2)	−7 (1)	291 (41.7)	−4 (1)
ARB	103 (17.9)	**M:** −4 (1) **W:** −4 (1)	179 (34.1)	**M:** −7 (1) **W:** −3 (1)	245 (48.0)	**M:** −8 (1) **W:** −7 (1)
Others	47 (27.8)	−10 (1)	73 (36.4)	−10 (1)	57 (35.8)	−8 (1)

Data presented as mean difference with standard error (SE), estimates weighted to represent the US population. Means are adjusted for age, gender, ethnicity, health insurance status, smoking status, physical activity, and education. ACE = angiotensin-converting enzyme; CCB = calcium channel blocker; ARB = angiotensin receptor blocker; SBP = systolic blood pressure; DBP = diastolic blood pressure; BMI = body mass index; normal weight = 18.5 kg/m^2^ < BMI < 25 kg/m^2^; overweight = 25 kg/m^2^ ≤ BMI < 30 kg/m^2^; obesity = BMI ≥ 30 kg/m^2^; M = men; W = women. ^*∗*^ Significantly different from normal weight (*P* < 0.05); ^†^significantly different from overweight (*P* < 0.05).

## Data Availability

The NHANES dataset used to support the findings of this study are available from CDC at https://www.cdc.gov/nchs/nhanes/index.htm.
